# The HIV/AIDS response as we knew it is over: Where do we go from here?

**DOI:** 10.1002/jia2.70077

**Published:** 2026-02-20

**Authors:** Chris Beyrer, Jirair Ratevosian, Tom Carpino, Nora E. Rosenberg, Huub C. Gelderblom, Patrick S. Sullivan, Steve G. Deeks, Glenda Gray

**Affiliations:** ^1^ Duke Global Health Institute Durham North Carolina USA; ^2^ UNC Gillings School of Global Public Health Chapel Hill North Carolina USA; ^3^ Fred Hutch Cancer Center Seattle Washington USA; ^4^ Rollins School of Public Health Emory University Atlanta Georgia USA; ^5^ University of California San Francisco California USA; ^6^ South African Medical Research Council Cape Town South Africa; ^7^ Infectious Disease and Oncology Research Institute University of Witwatersrand Johannesburg South Africa

**Keywords:** AI, cure, HIV, incidence, number needed to treat, PEPFAR, prevention

## Abstract

**Introduction:**

The global HIV response, once a model of progress and innovation, faces a profound moment. Despite four decades of pivotal scientific and programmatic advances—most notably in antiretroviral therapy (ART) and pre‐exposure prophylaxis (PrEP)—the world remains off track to meet the 2025 and 2030 targets for ending AIDS as a public health threat. New acquisitions and AIDS‐related deaths remain unacceptably high, particularly among key populations and in low‐ and middle‐income countries. The abrupt U.S. funding reversals in 2025 have severely disrupted support for HIV efforts. Cuts to U.S. and international institutions have compromised HIV prevention, treatment and surveillance systems worldwide, and may already have begun reversing two decades of progress.

**Discussion:**

To avert this crisis, the HIV and public health community, together with governments and global funders, must urgently invest in scaling long‐acting treatment and prevention tools, rebuild disaggregated data systems and strengthen implementation science rooted in community‐led approaches. Digital health technologies offer promise to enhance service delivery, surveillance, monitoring and evaluation, especially in resource‐constrained settings, but demand ethical governance and infrastructure investment. The global research ecosystem must become more evenly distributed and inclusive, with a shift towards country‐led partnerships, national data sovereignty and regional co‐operation.

**Conclusions:**

Looking to 2030 and beyond, the strategy to end HIV should include expanded access to long‐acting ART and PrEP, sustained investments in HIV vaccine and cure research, and robust monitoring and evaluation systems. Achieving epidemic control—and ultimately ending the HIV pandemic—will require not only biomedical tools but also political will, community leadership and equitable financing. The lessons of the past underscore that sustained progress is possible, but only if we meet this moment with urgency, imagination and solidarity.

## 1 INTRODUCTION

The global response to the HIV pandemic is one of the greatest successes in modern health, transforming a uniformly fatal infection to global delivery of safe, effective therapies [1] to over 70% of persons living with HIV worldwide. Behavioural and biomedical prevention science advances have significantly reduced new acquisitions; innovations in HIV treatment and prevention have accelerated since the demonstration that antivirals could be used for the prevention of HIV acquisitions [1]. But in 2024, the global HIV response was off track to meet the 2025 and 2030 UNAIDS goals for reductions in mortality or incident infections [2]. In a July 2024 report, UNAIDS described two decades of sustained progress, marked expansion of treatment and reductions in deaths and new acquisitions—largely due to U.S. funding [3]. However, new acquisitions were more than triple the 2025 target; AIDS‐related deaths were more than double the 2025 target. UNAIDS still contended that bold leadership could still achieve the ambitious global goal of ending AIDS as a public health threat by 2030.

Oral pre‐exposure prophylaxis (PrEP) start rates were increasing in 2023 and 2024, but the public health benefits of this strategy were limited by the poor adherence, retention and persistence, except among gay and bisexual men in high treatment coverage settings [4, 5]. Incidence data from the placebo arms of multiple HIV prevention trials continued to show sustained high rates of incident infections in cisgender women in sub‐Saharan Africa and among MSM [2]. Taken together, these realities suggested that epidemic control would require both an intensified focus on testing and treating all people living with HIV and an increased focus on primary prevention, including the rollout of oral PrEP and new long‐acting injectable PrEP agents.

The field has continued to innovate, as more options for treatment and prevention emerge. The efficacy of long‐acting PrEP—injectable cabotegravir [6] and lenacapavir [7, 8]—has been remarkable, with nearly 100% efficacy found in clinical trials, even among women. There was renewed hope that ending HIV as a public health threat might indeed be achievable by 2030, and ongoing understanding that this would require substantial increases in prevention funding and programming to become a reality.

However, the impact of these remarkable scientific advances has been uneven. The early implementation of ART in Africa resulted in rapid reductions in mortality and associated broad societal benefits, including improved economies and more stable social systems. HIV‐specific mortality rates are now lower, but have stabilized and remain high for many communities. Mortality rates are expected to rise unless countries intensify their responses to advanced HIV disease, which now accounts for a disproportionate share of AIDS‐related deaths—even in high‐income settings with high ART coverage [9].

The limitations of the global response are even more pronounced in the paediatric population. Among children, the rates of ART uptake are much lower than among adults [10]. Adherence challenges persist through childhood, adolescence and early adulthood. Mortality rates remain much higher across these ages compared to adults.

As of late 2024, the global response to HIV could best be described as a massive but incomplete success story. We were approaching the level of response needed to end the epidemic, despite uneven progress in some key populations. On the central metrics—incident infections and mortality—we were still far from the 2030 UNAIDS goals, with limited year‐to‐year improvements. A renewed global response will be essential to ending the epidemic.

## 2 THE 2025 INFLECTION POINT IN U.S. GLOBAL HEALTH POLICY

In 2025, the promise of continued progress was disrupted by sweeping policy and funding shifts in the United States, the largest bilateral HIV donor in the world [11]. Within the first month of the new administration, the Department of Government Efficiency issued dozens of executive orders targeting gender identity, foreign aid and global health programming. The U.S. President's Emergency Plan for AIDS Relief (PEPFAR) was initially affected by cuts, but later received a partial waiver for treatment programmes and prevention of mother‐to‐child transmission. These massive and rapid shocks to the system have resulted in sustained political, financial and ideological disruptions—disproportionately affecting communities impacted by HIV and the programmes and partnerships designed to support them [12, 13].

Within the United States, seven of 15 branches of the Division of Global HIV and tuberculosis were eliminated. The destruction of the U.S. Agency for International Development (USAID) resulted in a dramatic reduction in force [14]. The U.S. withdrawal on public health has catalysed the reorganization and downsizing of the World Health Organization and UNAIDS. Global and national research and surveillance systems are at risk, which will result in severe limitations in understanding disease transmission dynamics. Projects supporting surveillance in the United States (e.g. the US National Health Behavioral Surveillance System [15]), and internationally (e.g. Population‐Based HIV Impact Assessments [16]), are at risk of or have already lost funding.

The erosion of U.S. surveillance capacity threatens domestic epidemic control and global epidemic intelligence: U.S.‐backed systems have long underpinned response coordination and cross‐national research. Further, the PEPFAR waiver excluded funding for HIV prevention beyond programmes for pregnant and breastfeeding women and halted the collection and reporting of data disaggregated by key populations [14]. This is especially concerning given that 91% of global PrEP initiations were supported by PEPFAR in 2024, making access highly vulnerable to shifts in donor priorities [17]. Eliminating disaggregated data on gender identity and key populations will weaken efforts to deliver and evaluate targeted prevention across PEPFAR‐supported countries.

HIV prevention and treatment pipelines face interruptions across the globe. The cuts to HIV treatment and prevention may result in significant increases in new acquisitions, HIV‐related deaths and medical care costs [18, 19]; the actual impact will depend on how effectively countries and global programmes fill emerging gaps. Cuts to PEPFAR and international aid are projected to result in 4–11 million new HIV acquisitions in low‐ and middle‐income countries [18], many times the current annual 1.3 million new HIV acquisitions globally. Similarly, even modest reductions in U.S. PrEP coverage are estimated to result in thousands of preventable HIV acquisitions and billions of dollars of excess HIV treatment costs [19]. Countries with large numbers of people in need of HIV treatment have prioritized treatment over prevention, but cutting prevention means that future treatment cost burdens are likely to rise.

Dismantling long‐standing HIV U.S./African research collaborations threatens global progress [20]. The Fogarty International Center is slated for elimination under the U.S. President's FY26 budget proposal [21]. The NIH's new policy requiring direct federal oversight of foreign subawards introduces significant administrative burdens, erodes trust and slows implementation of joint research [22]. Some international grants and clinical trials have been terminated prematurely, cutting off participants from lifesaving treatment, weakening critical global partnerships and losing clinical trial data to inform better treatments and prevention strategies.

The HIV research pipeline also faces growing threats from sharp reductions in training and career development programmes (e.g. NIH T and F awards, D43 grants and diversity supplements)—programmes that have historically supported the next generation of HIV researchers and global health leaders. With uncertain future funding, early career scientists may leave the field altogether, further jeopardizing the continuity and innovation of HIV research.

## 3 DISCUSSION

### 3.1 The new reality: where are we going?

In the wake of policy and funding upheavals, many countries are now contending with significant fallout. Trust in public institutions and global donors has eroded, particularly among communities most affected by HIV. Recent U.S. actions point to a partial stabilizing of these shocks. The State Department's America First Global Health Strategy—shifting towards bilateral compacts—signals a major restructuring [23], while the administration's multi‐year pledge to the Global Fund's eighth replenishment provides a measure of stability amid uncertainty. These steps do not reverse the earlier disruptions, but they do create limited areas of continuity that countries are now recalibrating around.

Rebuilding this trust must be a cornerstone of any renewed global HIV response (Table 1). We must restore relationships with organizations working with key population groups, ensure transparency and predictability in data and resource flows, and ground donor‐led prevention and treatment programmes in national leadership. At the same time, new models of academic collaboration and funding are needed. The global HIV research ecosystem must pivot from siloed, Northern‐dominated structures to more distributed, agile and inclusive networks led by scientists in the Global South. In‐country government funding for local research will address this disparity in middle‐income countries, such as South Africa, and political commitment to increase research funding to at least 2% of the total public sector health expenditure will pave the way for empowered participation in research partnerships. These partnerships must prioritize innovation and extend to implementation, evaluation and systems‐building to promote data sovereignty [24].

**Table 1:** Recommendation to sustain the global HIV/AIDS response

Recommendation	Solution
Scale long‐acting HIV prevention and treatment	Rapidly scale long‐acting ART and PrEP through aligned regulation, procurement and delivery systems that can also support future cure and vaccine platforms.
Rebuild and protect HIV surveillance and data systems	Restore disaggregated surveillance and monitoring systems, including key population data, to guide programmes and track equity and impact.
Strengthen community‐led HIV responses	Protect and diversify support for community‐ and key population–led organizations to sustain rights‐based prevention and treatment services.
Shift to country‐led and equitable research partnerships	Reorient HIV research towards Global South leadership, national financing, data sovereignty and regional collaboration.
Integrate digital health and AI responsibly	Use AI‐enabled tools to improve service delivery and surveillance, alongside investments in governance, infrastructure and community engagement.
Sustain combination prevention strategies	Maintain layered prevention approaches to preserve choice and effectiveness as new tools scale unevenly.
Develop innovative and equitable financing models	Expand innovative purchasing, licensing and pricing models, especially for middle‐income countries.
Reinvigorate HIV vaccine research	Sustain political and financial support for HIV vaccine development and counter disinformation.
Keep HIV cure research central to global strategy	Continue investment in scalable cure research to reduce long‐term dependence on lifelong ART.
Rebuild trust and global solidarity	Anchor renewed HIV efforts in transparency, shared accountability and co‐designed partnerships.

Considering the abrupt forfeiture of U.S. leadership, it is critical that countries and communities increasingly own, control and benefit from the data that shape their health futures [25]. Rather than perpetuating top‐down aid structures, donors should embrace models rooted in co‐investment, shared accountability and mutual benefit. National governments, communities and donors must co‐design strategies that reflect local priorities and jointly steward progress. Encouragingly, efforts led by PEPFAR, Global Fund and UNAIDS to support country ownership, data transparency and inclusive planning offer useful models to reimagine what true partnership can look like [26]. More recently, the Kigali Call to Action, endorsed by the International AIDS Society, charted a way forward towards global HIV solidarity, emphasizing shared accountability and aligning closely with the Accra Accord's call for health sovereignty, local leadership and mutually beneficial partnerships [27].

Embracing the potential of emerging digital technologies offers new avenues for accelerating progress in the HIV response. Artificial intelligence (AI) presents a promising set of opportunities to enhance HIV surveillance and programming by improving efficiency, personalization and system reach—including in resource‐constrained settings [28]. Embedded within equitable and well‐designed health systems, AI‐powered tools have shown, in limited settings, to streamline service delivery through automated triage, adherence support, virtual consultations and predictive analytics [29]. These innovations have the potential to support existing surveillance, treatment and prevention goals. Achieving scalable and ethical integration of AI into HIV programmes will require sustained investments in infrastructure, digital literacy, data management and meaningful community engagement.

Biomedical innovation remains essential to advancing HIV prevention and treatment. New tools like long‐acting injectable PrEP, implants and extended‐release oral formulations offer critical alternatives for individuals unable or unwilling to take daily oral PrEP. Scaling up lenacapavir not only expands the prevention toolkit but also establishes the infrastructure—clinical, regulatory and programmatic—needed to support future innovations, including HIV cure strategies that may share delivery mechanisms or adherence requirements.

Going forward, new interventions will require innovative financing models, especially in the high‐middle income settings, like subscription‐based drug purchasing mechanisms. Broadening access to generics will also require stronger engagement from the private sector, philanthropic actors and global research networks to drive product development, equitable distribution and real‐time monitoring. For new biomedical modalities, this includes expanding licensing arrangements, favourable pricing schemes and innovative funding mechanisms for middle‐income countries restricted from company licensing arrangements.

The pivot towards long‐acting PrEP should not obscure the centrality of combination prevention. Countries that achieved significant declines in HIV incidence did so through layered strategies, including PrEP, treatment scale‐up, condom availability, sexually transmitted infection control, harm‐reduction services and accessible testing. With budgets tightening and access to novel prevention products uncertain, expanding choice across all effective prevention modalities becomes even more important.

To curb HIV incidence, PrEP will need to reach tens of millions of people worldwide—especially key populations at highest risk and high‐incidence African settings. UNAIDS estimates that epidemic control will require scaling to at least 20 million person‐years of PrEP by 2030; today's uptake targets are a fraction of that target [30]. Modelled estimates from 15 African countries suggest that 25 million women and 30 million men would need access to highly effective PrEP to avert two‐thirds of new acquisitions [31, 32]. Reaching this scale will require coordinated investments in demand forecasting, regulatory alignment, procurement systems and community‐driven demand generation. Strategic early investments in long‐acting PrEP delivery can accelerate access while building a foundation for future biomedical breakthroughs—including a functional cure.

Support is also needed for community‐led organizations, particularly those led by key populations who continue to face political harassment, restrictions or closure, often exacerbated when U.S. diplomatic or financial presence diminishes. Protecting this response will require diversified support from regional bodies, philanthropic actors and multilateral institutions; legal and political advocacy to defend civic space; and investment in organizational resilience independent of donor volatility. Without a robust key population–led civil society, no combination of biomedical advances can sustain epidemic control.

In generalized epidemic settings, where nearly all sexually active individuals are at risk for HIV, the scale of prevention may still be inadequate to curb the epidemic in the long‐term. Delivering PrEP to an entire society might never be possible. In these contexts, only a safe, effective and scalable HIV vaccine can offer long‐term epidemic control. However, the HIV vaccine field now faces mounting political threats, particularly against mRNA‐based research platforms. Revitalizing scientific support, countering disinformation about vaccines and marshalling political support for HIV vaccine development is an urgent priority.

The need to provide lifelong ART to over 40 million people underscores the fragility of current delivery systems. The global population of people living with HIV will reach 50 million by 2050; the case for a scalable cure is urgent. After over a decade of sustained investment—largely led by the NIH—we see progress towards post‐ART control or remission. Promising “one‐shot cure” approaches are under investigation in non‐human primates, and early stage human trials are on the horizon. A safe, affordable and scalable HIV cure must remain a central pillar of global HIV strategy [33]. Relatedly, success across prevention, treatment, cure and vaccines depends on robust, real‐time data systems, making the rebuilding and resourcing of monitoring and evaluation essential to guide—not just count—progress.

**Figure 1:**
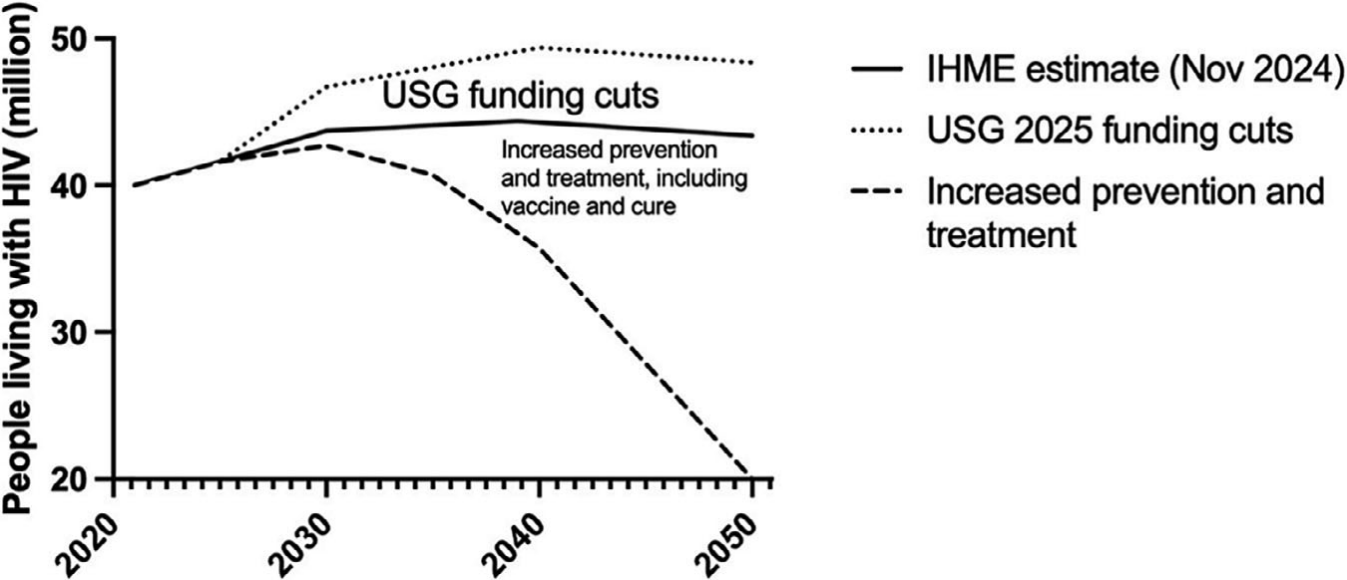
Projected HIV outcomes under three global funding scenarios (adapted from Ref. [34]).

We should look towards 2050 with a focus on ending the HIV pandemic through a coordinated, multipronged strategy [34]. The choices donors and governments make now will shape the fate of the HIV response for decades to come (Figure 1). Combined and at scale, these tools are essential—and sufficient—for epidemic control. If vaccines and cures come online, the demand for ART and PrEP might eventually decline—but only if systems are in place to ensure safe, equitable and effective integration. Throughout this transition, rigorous implementation science and data stewardship will maximize impact and sustain global progress.

## 4 CONCLUSIONS

We have been here before. We have lived through denial, underfunding and backlash. And each time, the global HIV response found its footing again—anchored by science, grounded in communities and powered by a coalition that refuses to give up. The question before us is not whether continued progress is possible, but whether we are willing to meet this moment with the urgency and imagination it demands.

What the past decades have shown is that success depends not only on biomedical tools, but on the systems that deliver them and the people they serve. Community leadership, political will and sustained financing are not ancillary—they are foundational. These pillars enabled countries with limited resources to expand ART access, integrate prevention services and reduce mortality. They proved that with the right approach, epidemic control is achievable—even in the most challenging settings. Today, those same pillars are at risk. And so too is the progress we have made.

The road ahead requires more than returning to business as usual. It calls for a renewed commitment to equity, to innovation and to global solidarity. That means investing in long‐acting tools with a sustained remission approach, building data sovereignty and accountability into national systems, and protecting the rights and dignity of those most affected. It also means resisting complacency. The HIV response taught the world how to build effective, inclusive global health infrastructure. We must now apply those lessons—urgently, unapologetically and together.

## COMPETING INTERESTS

No conflicts of interest to declare.

## AUTHORS’ CONTRIBUTIONS

CB conceptualized the paper, defined the scope and led the overall framing and direction of the manuscript. JR, NER, GG, SGD and PSS further expanded the manuscript. All authors contributed substantively to the literature review, critical analysis and interpretation of the findings.

## Data Availability

Data sharing is not applicable to this article, as no datasets were generated or analysed during the current study.

## References

[1] Bekker LG , Beyrer C , Mgodi N , Lewin SR , Delany‐Moretlwe S , Taiwo B , et al. HIV infection. Nat Rev Dis Primers. 2023;9(1):42. 37591865 10.1038/s41572-023-00452-3

[2] Beyrer C , Tomaras GD , Gelderblom HC , Gray GE , Janes HE , Bekker LG , et al. Is HIV epidemic control by 2030 realistic? Lancet HIV. 2024;11(7):e489–e494. 38925732 10.1016/S2352-3018(24)00098-5 PMC11416730

[3] New UNAIDS report shows AIDS pandemic can be ended by 2030, but only if leaders boost resources and protect human rights now [Internet]. [cited 2025 June 11]. Available from: https://www.unaids.org/en/resources/presscentre/pressreleaseandstatementarchive/2024/july/20240722_global‐aids‐update

[4] Grulich AE , Kaldor JM . Trends in HIV incidence in homosexual men in developed countries. Sex Health. 2008;5(2):113–118. 18588775 10.1071/sh07075

[5] HIV Transmission Elimination AMsterdam (H‐TEAM) Initiative. A 95% decline in estimated newly acquired HIV infections, Amsterdam, 2010 to 2022. Euro Surveill. 2023;28(40):2300515. Available from: 10.2807/1560-7917.ES.2023.28.40.2300515 37796442 PMC10557385

[6] Landovitz RJ , Donnell D , Clement ME , Hanscom B , Cottle L , Coelho L , et al. Cabotegravir for HIV prevention in cisgender men and transgender women. N Engl J Med. 2021;385(7):595–608. 34379922 10.1056/NEJMoa2101016 PMC8448593

[7] Kelley CF , Acevedo‐Quiñones M , Agwu AL , Avihingsanon A , Benson P , Blumenthal J , et al. Twice‐yearly lenacapavir for HIV prevention in men and gender‐diverse persons. N Engl J Med. 2025;392(13):1261–1276. 39602624 10.1056/NEJMoa2411858

[8] Bekker LG , Das M , Abdool Karim Q , Ahmed K , Batting J , Brumskine W , et al. Twice‐yearly lenacapavir or daily F/TAF for HIV prevention in cisgender women. N Engl J Med. 2024;391(13):1179–1192. Available from: https://www.nejm.org/doi/abs/10.1056/NEJMoa2407001 39046157 10.1056/NEJMoa2407001

[9] Trickey A , Ambia J , Glaubius R , van Schalkwyk C , Imai‐Eaton JW , Korenromp EL , et al. Excess mortality attributable to AIDS among people living with HIV in high‐income countries: a systematic review and meta‐analysis. J Int AIDS Soc. 2024;27(11):e26384. 39496514 10.1002/jia2.26384 PMC11534483

[10] Joint United Nations Programme on HIV/AIDS . UNAIDS. Global AIDS Strategy 2026–2031. UNAIDS; 2025.

[11] Joint United Nations Programme on HIV and AIDS (UNAIDS) . Donor government funding for HIV in low‐ and middle‐income countries in 2024. Kaiser Family Foundation; 2025.

[12] Matanje B , Masha RL , Rwibasira G , Ngure K , Yahaya HB , Anam FR , et al. The global HIV response at a crossroads: protecting gains and advancing sustainability amid funding disruptions. Lancet HIV. 2025;12(7):e532–e536. Available from: 10.1016/S2352-3018(25)00106-7 40354797

[13] Ratevosian J , Millett G , Honermann B , Bennett S , Connor C , Bekker LG , et al. PEPFAR under review: what's at stake for PEPFAR's future. Lancet. 2025;405(10479):603–605. 39929219 10.1016/S0140-6736(25)00258-2

[14] Kaiser Family Foundation (KFF) . U.S. Foreign Aid Freeze & Dissolution of USAID: Timeline of Events. 2025. KFF, Accessed November 30, 2026 Available from: https://www.kff.org/global‐health‐policy/u‐s‐foreign‐aid‐freeze‐dissolution‐of‐usaid‐timeline‐of‐events/

[15] Gallagher KM , Sullivan PS , Lansky A , Onorato IM . Behavioral surveillance among people at risk for HIV infection in the U.S.: the National HIV Behavioral Surveillance System. Public Health Rep. 2007;122(Suppl 1):32–38. 17354525 10.1177/00333549071220S106 PMC1804113

[16] Gaumer G , Senthil Kumar VS , Crown W , Jordan M , Hurley C , Subramanian M , et al. Equity of the HIV epidemic response in 13 African countries. Afr J AIDS Res. 2023;22(4):276–289. 38117740 10.2989/16085906.2023.2277887

[17] U.S. Department of State . PEPFAR — World AIDS Day 2024. 2024. Accessed November 14, 2025 Available from: https://www.state.gov/the‐united‐states‐president‐s‐emergency‐plan‐for‐aids‐relief/pepfar‐world‐aids‐day‐2024/

[18] Brink DT , Martin‐Hughes R , Bowring AL , Wulan N , Burke K , Tidhar T , et al. Impact of an international HIV funding crisis on HIV infections and mortality in low‐income and middle‐income countries: a modelling study. Lancet HIV. 2025;12(5):e346–e354. 40157378 10.1016/S2352-3018(25)00074-8

[19] Sullivan PS , Wall KM , Juhasz M , DuBose S , Crowley JS , Breyer C , et al. Excess HIV infections and costs associated with reductions in HIV prevention services in the US. JAMA Netw Open. 2025;8(9):e2531341. 40932715 10.1001/jamanetworkopen.2025.31341 PMC12426795

[20] Ratevosian J , Beyrer C , Chola M , Huchko M , Machingaidze S , Udayakumar K , et al. The new U.S. Global Health Strategy — a reset of America's health cooperation. N Engl J Med. 2025;393(22):2180–2182. 41324249 10.1056/NEJMp2514898

[21] People O . White House releases FY26 budget request [Internet]. KFF; 2025 [cited 2025 Nov 24]. Available from: https://www.kff.org/global‐health‐policy/fact‐sheet/white‐house‐releases‐fy26‐budget‐

[22] NIH . Updated NIH Policy on Foreign Subawards. Notice Number NOT‐OD‐25‐104. National Institutes of Health; 2025. Accessed January 23, 2026. https://grants.nih.gov/grants/guide/notice-files/NOT‐OD‐25‐104.html.

[23] Corey L , Ratevosian J , Beyrer C , Currier J , Eron J , Cohen MS , et al. How HIV research drives health innovation in multiple diseases. Nat Med (Baltimore). 2025;31(12):3965–3967. 10.1038/s41591-025-04019-5. PMC13441067 41136629

[24] Chola M , Sikazwe I , Robalo M , Oduro‐Bonsrah P , Coutinho A , Sheneberger R , et al. Africa's defining moment: the time to lead the HIV response is now. Lancet Glob Health. 2025;13(5):e801–e802. 40086462 10.1016/S2214-109X(25)00102-0

[25] Kaseya J . Africa's health security and sovereignty agenda: a new way forward. Lancet. 2025;406(10518):2394–2396. 41265464 10.1016/S0140-6736(25)02315-3

[26] HIV response sustainability roadmap part A: companion guide. Geneva: UNAIDS; 2024.

[27] Chola M , Ratevosian J , Auerbach JD . Recommitting to global solidarity: introducing the Kigali Call to Action. Lancet. 2025;406(10502):439. 10.1016/S0140-6736(25)01427-8 40690923

[28] Ratevosian J , Reid M , Ni Z , et al. Reimagining HIV prevention with artificial intelligence. Lancet HIV. 2025;12(10):e670–e671. 10.1016/S2352-3018(25)00158-4 40516546

[29] Cheah MH , Gan YN , Altice FL , Wickersham JA , Shrestha R , Salleh NAM , et al. Testing the feasibility and acceptability of using an artificial intelligence chatbot to promote HIV testing and pre‐exposure prophylaxis in Malaysia: mixed methods study. JMIR Hum Factors. 2024;11:e52055. 38277206 10.2196/52055 PMC10858413

[30] UNAIDS. Services that save the lives of people living with HIV. UNAIDS Global AIDS Update. United Nations; 2025.

[31] Rosenberg NE , Shook‐Sa BE , Young AM , Zou Y , Stranix‐Chibanda L , Yotebieng M , et al. A human immunodeficiency virus type 1 risk assessment tool for women aged 15–49 years in African countries: a pooled analysis across 15 nationally representative surveys. Clin Infect Dis. 2024;79(5):1223–32. 38657086 10.1093/cid/ciae211 PMC11581698

[32] Rosenberg NE , Young AM , Zou Y , et al. An HIV‐1 risk assessment tool for men aged 15–59 years in 13 African Countries: A pooled analysis of Nationally Representative Surveys. J Acquir Immune Defic Syndr. 2026;101(2):173–182. 10.1097/QAI.0000000000003773. 41065287 PMC13009710

[33] Deeks SG , Archin N , Cannon P , Collins S , Jones RB , de Jong MAWP , et al. Research priorities for an HIV cure: International AIDS Society Global Scientific Strategy 2021. Nat Med. 2021;27(12):2085–98. 34848888 10.1038/s41591-021-01590-5

[34] GBD 2021 HIV Collaborators . Global, regional, and national burden of HIV/AIDS, 1990–2021, and forecasts to 2050, for 204 countries and territories: the Global Burden of Disease Study 2021. Lancet HIV. 2024;11(12):e807–e822. 39608393 10.1016/S2352-3018(24)00212-1 PMC11612058

